# Correlation of Patient-Reported Outcome (PRO-2) with Endoscopic and Histological Features in Ulcerative Colitis and Crohn's Disease Patients

**DOI:** 10.1155/2020/2065383

**Published:** 2020-04-02

**Authors:** Sanja Dragasevic, Aleksandra Sokic-Milutinovic, Milica Stojkovic Lalosevic, Tamara Milovanovic, Srdjan Djuranovic, Ivan Jovanovic, Sanja Rajic, Mirjana Stojkovic, Biljana Milicic, Stefan Kmezic, Branislav Oluic, Marko Aleksic, Aleksandra Pavlovic Markovic, Dragan Popovic

**Affiliations:** ^1^Clinic for Gastroenterology and Hepatology, Clinical Center of Serbia, Koste Todorovica 2, 11000 Belgrade, Serbia; ^2^School of Medicine, University of Belgrade, Dr Subotica 8, 11000 Belgrade, Serbia; ^3^Institute for Medical Informatics, University of Belgrade, School of Dentistry, Rankeova 4, Belgrade, Serbia; ^4^Institute for Orthopedic Surgery “Banjica”, 28 Mihaila Avramovića Street, Belgrade, Serbia

## Abstract

**Methods:**

A cross-sectional study was conducted in consecutive newly diagnosed patients with inflammatory bowel disease in a tertiary care referral center. The initial evaluation included patient-reported outcome for stool frequency subscore and rectal bleeding. Endoscopic activity was determined using the Mayo scoring system for ulcerative colitis and the Simple Endoscopic Score for Crohn's disease. Histopathological activity was assessed using a validated numeric scoring system.

**Results:**

We included 159 patients (63 Crohn's disease with colonic involvement and 96 with ulcerative colitis). We found significant correlation between the Mayo endoscopic subscoring system and histology activity in ulcerative colitis, while no correlation was found in patients with Crohn's disease. Patient-reported outcome showed inverse correlation with endoscopic and histological activity in Crohn's disease (*r*_s_ = −0.67; *r*_s_ = −0.72), while positive correlation was found in ulcerative colitis (*r*_s_ = 0.84; *r*_s_ = 0.75). *Interpretation and Conclusions*. Patient-reported outcome is a practical and noninvasive tool for assessment of disease activity in ulcerative colitis patients but not in Crohn's disease.

## 1. Introduction

Assessment of inflammatory bowel disease (IBD) activity is necessary in order to determine adequate therapeutic approach for these patients. In everyday clinical practice, assessment is based on laboratory analyses, endoscopic and histological evaluation, and the patient's reported outcome (PRO) [1–4].

Because IBD is a chronic inflammatory disease with periods of relapse and remission, disease monitoring using clinical activity indexes is of great importance. Active inflammatory bowel disease in both ulcerative colitis (UC) and Crohn's disease (CD) with colonic involvement includes, as per definition, presence of rectal bleeding (RB) and diarrhoea. In recent years, several questionnaires have been developed, bearing in mind that PRO represents an important endpoint and major therapeutic goal of IBD [5]. Furthermore, in order to avoid unpleasantness of bowel preparation and repeated ileocolonoscopies, as well as to avoid additional costs, noninvasive methods as well-designed questionnaires are required in monitoring the disease activity. Previously published studies suggested discrepancies between PRO and endoscopic and histological disease activity in patients with UC [6–9]. Studies conducted in CD patients investigated a two-item PRO (PRO-2) with stool frequency (SF) and abdominal pain (AP), while a three-item PRO (PRO-3) included additional general well-being [5, 10].

Endoscopic activity of IBD is assessed using previously validated endoscopic scores. The endoscopic scoring system is designed as an objective tool for monitoring and measuring severity of IBD activity [11, 12]. The Mayo scoring system (MS) is the most frequently used endoscopic activity score designed for UC and consists of the assessment of granulation scattering, vascular pattern, vulnerability, and mucosal damage [12, 13]. The Simple Endoscopic Score for Crohn's disease (SES-CD) takes into account four parameters (presence of ulcers, percentage of ulcerated surfaces, affected surface, and presence of strictures) that need to be scored in five bowel segments (the rectum, sigmoid and left colon, transverse colon, right colon, and ileum) [14, 15].

Additionally, histological activity is based on the analysis of architectural abnormalities and presence of inflammatory features. In UC, histological examination of mucosa may reveal crypt distortion, atrophy, Paneth metaplasia, and diffuse mucosal inflammatory infiltrate, while basal plasmacytosis could suggest a severe chronic inflammation [16, 17]. Histological changes in CD are usually focal and discontinuous with chronic active mucosal inflammation along transmural lymphoid hyperplasia. Sometimes the architectural distortion of CD can be similar to UC, while granuloma can be detected in some, but not all, CD patients [16]. Histological scoring systems rely on the analysis of haematoxylin and eosin- (H&E-) stained sections using a stepwise or numerical system [16, 18]. The most widely used score for UC is the Geboes score, a six-grade classification system for inflammation (0: structural change only, 1: chronic inflammation, 2: neutrophils in the lamina propria, 3: neutrophils in the epithelium, 4: crypt destruction, and 5: erosions or ulcers) [18].

The aim of our study was to determine possible association between a two-item PRO based on stool frequency and rectal bleeding (PRO-2) and endoscopic and histological activity in patients with ulcerative colitis and colonic Crohn's disease.

## 2. Materials and Methods

### 2.1. Patients

We conducted a cross-sectional study at the clinic for gastroenterology and hepatology, Clinical Centre of Serbia, Belgrade, in 159 newly diagnosed IBD patients (96 UC and 63 CD with colonic involvement) from 2016 until 2018. In all patients, IBD was diagnosed using clinical, endoscopic, radiological, and histological criteria for IBD diagnosis. We excluded patients with CD ileitis (L1), proximal disease location (L4), and indeterminate IBD. This study was approved by the Institutional Review Board and conducted in accordance with the Helsinki Declaration after patients signed written informed consent.

### 2.2. Patient-Reported Outcome Measures in Inflammatory Bowel Disease

The patient-reported outcome (PRO-2) included evaluation of stool frequency (SF) and a rectal bleeding (RB) subscore on 3 consecutive days in a week before colonoscopy, excluding the day when bowel cleaning for colonoscopy was performed. The SF subscore was graded as follows: scores 0 (for normal number of stools), 1 (1-2 stools more than normal), 2 (3-4 stools more than normal), and 3 (5 or more stools more than normal). The RB subscore was determined with values 0 (no blood seen), 1 (streaks of blood with the stool less than half the time), 2 (obvious blood with the stool most of the time), and 3 (blood alone passed).

### 2.3. Endoscopic Evaluation of Patients with Inflammatory Bowel Disease

All ileocolonoscopies were performed by two experienced gastroenterologists using Olympus high-definition colonoscopes. Endoscopic disease activity was reported using endoscopic scoring systems (Mayo scoring system for UC and Simple Endoscopic Score for CD). Inactive ulcerative colitis disease was graded as Mayo score 0/1, while the SES-CD index was interpreted as follows: score less than 2 indicated inactive, 3-6 mildly active, 7-16 moderately active, and over 16 severely active disease.

### 2.4. Histological Assessment of Inflammatory Bowel Disease Activity

Histological activity was assessed using the Geboes grading system. The score ranged from 0 to 5.4, with higher scores indicating more severe histological inflammation [6]. Although target biopsies were taken from regions of endoscopic activity in CD patients and assessed for histological activity, the use of histological scoring in Crohn's disease was limited by discontinuous bowel involvement.

### 2.5. Theory

Further studies to determine validated PRO accuracy and utility in the assessment of IBD activity are needed for comprehensive research in which correlation between patients' reported symptoms and endoscopic and histological disease activity will be analysed on clinically well-characterized subgroups of IBD patients. A “more personalized” approach including patients' reported symptoms in a validated PRO could be beneficial for more efficient management of the disease and better prognosis.

### 2.6. Calculation

Statistical analyses were performed using SPSS software version 23 (Chicago, IL). Descriptive data were expressed as mean values, standard deviations, median, and range for continuous measures or percentage of a group for discrete measures. Numeric data were tested for normal distribution using the Kolmogorov-Smirnov test. One-way ANOVA and the Kruskal-Wallis test were used to assess differences between groups. Categorical data were analysed using the Pearson chi-square test. The correlation between the observed parameters was analysed using Spearman's correlation coefficients. *p* < 0.05 was required to reject the null hypothesis.

## 3. Results

Our study included 159 IBD patients (96 with ulcerative colitis and 63 with Crohn's disease). Patients' characteristics are summarized in Table 1.

### 3.1. Evaluation of Endoscopic and Histological Measures in Study Population

No significant differences were found between patients with different disease extension of UC in respect of MS and histological activity (Table 2) (*p* = 0.641 and *p* = 0.595, respectively). The Mayo endoscopic score correlated with the degree of histological activity (Spearman coefficient *r*_s_ = 0.914) regardless of the disease localization (Table 3). All patients with CD included in the study had SES − CD score > 2 (active disease) and mean value 13.50 ± 10.82 for colitis and 19.35 ± 14.61 for ileocolitis. Although SES-CD was higher in patients with more extensive disease, no correlation was observed between SES-CD and the histological activity of disease regardless the disease localization (Table 4).

### 3.2. Patient-Reported Outcome Assessment

Tables 5 and 6 summarize the relationship of symptom scores SF and RB with endoscopic activity score in the CD and UC groups. Subscores of SF, RB, and SF+RB in comparison with MS showed higher values of sensitivity, specificity, and positive predictive value (PPV) than in cases with CD patients and the SES-CD score. Statistically significant association was found between endoscopic and histopathological activities in ulcerative colitis with patient-reported outcome symptoms (*r*_s_ = 0.84 and *r*_s_ = 0.75, respectively). Nevertheless, we noticed that PRO showed inverse correlation with the SES-CD score and histological activity in Crohn's disease patients (*r*_s_ = −0.67 and *r*_s_ = −0.72, respectively).

## 4. Discussion

The Mayo endoscopic activity index is a practical tool, which could be used for objective assessment of mucosal healing in UC. However, results of our study have not shown such correlation between values of the SES-CD score and histological activity. Geboes and Dalle noticed that when the extent of disease needs to be defined, biopsies may double the yield of pathology compared to endoscopic findings of inflammation in IBD [18]. As reported by previous studies, variable results have been found comparing endoscopy scores and histological activity [2, 6]. In the study of Geboes et al., correlation between endoscopy and histology was found for endoscopically inflamed mucosa; however, data for mucosa without inflammation were inconsistent [17, 19]. Persistent microscopic lesions in the absence of endoscopic findings may indicate likelihood of relapse in ulcerative colitis [19]. In the review article by Travis et al., they suggested that microscopic healing in UC is a better predictor of durable remission and expected time to next relapse, when compared to endoscopy finding [19]. Indicators of acute mucosal inflammation were associated with increased risk of UC relapse [20, 21]. According to various multicentric studies, the use of the SES-CD score has been validated for the endoscopic activity assessment of Crohn's disease [12, 15]. In particular, the prospective study of Bryant et al. analysed data from 86 patients with Crohn's disease from 2005 until 2007 and found that SES-CD and Crohn's Disease Endoscopic Index of Severity (CDEIS) significantly correlate with the clinical activity of CD [15]. During the SES-CD index validation in the Daperno et al. study, significant correlation was reported between the index and the activity of CD [13].

As reported by consensus recommendations, endoscopic mucosal healing is defined as a resolution of visible inflammation and ulceration at endoscopy, variably Mayo 0 or 1 [17]. Often, endoscopic mucosal healing does not reflect quiescent microscopic disease. Numerous studies have demonstrated that histological inflammation persists in 16–100% of cases of endoscopically quiescent IBD [17, 21, 22]. The values of histopathological scores in IBD can often vary depending on the number, quality, and distribution of the samples taken. It is necessary to analyse the basal part of the mucosa, which is possible with cut biopsies, to allow the surface and the deep part of the biopsy to be identified in perpendicular sections [23]. Since the 1950s, many histopathological scores have been used for the disease activity assessment in UC. In the Mosli et al. systematic review, 22 histological scoring systems have been described for UC [16]. The first histological index of activity was described in 1956 by Truelove and Richards in a study that obtained 111 serial biopsy specimens from 42 UC patients [23]. One of the most widely used histopathological scores of disease activity in UC is the Geboes index which includes the assessment of architectural change, lamina propria neutrophils and eosinophils, neutrophils in the epithelium, crypt destruction, and erosion or ulceration [18].

In our study, the Geboes score was used and assigned to biopsies ranging from 0 to 5.4, with higher scores indicating more severe inflammation. However, there was no grading for basal plasmacytosis in the scoring system. The use of histological activity score in CD is limited by its segmentary nature, so target biopsies were required for the assessment, although transmural inflammation could only be analysed in resections. The validation of histological scoring in IBD remains challenging, and adequate assessment will require further regression analysis of specific histological features.

Osada et al. investigated the correlation between endoscopic and histological findings in 54 UC patients and found highly significant positive correlation in the region of the distal colon (rectum and sigma). But despite the positive correlation, their results showed that a site with endoscopic score 0 can often show evidence of histological disease activity [24]. According to the data of our study, there was no significant difference in endoscopic and histological activity scores related to distribution of the disease in UC patients. Previous studies have also suggested that the endoscopic appearance of IBD alone tends to underestimate the extent of disease relative to histological evaluation [21, 22].

However, endoscopic assessment of inflammation in CD has a better correlation with transmural inflammation than mucosal biopsy and thus the severity and extent of inflammation [15]. In concordance with previous studies, the discrepancy between endoscopy and histology findings is smaller in the case of active disease and greater for remissive IBD.

Recently, a “treat to target” approach has been proposed in IBD patients, and it includes a combination of PRO and endoscopic/histological assessments as treatment targets. Measures of PRO are based on standardized questionnaires reflecting the patient's perspective on their health condition [4, 25]. Colombel et al. found that symptom subscores in PRO correlate well with MS [5]. Nevertheless, presence of histological inflammation in the setting of endoscopic healing can be an underlying cause of clinical symptoms in IBD [26]. Inflammatory features in UC contribute to the changes in muscular organisation of the bowel wall reflecting structural and functional damage with absorptive modifications [26]. In CD, excessive deposition of extracellular matrix proteins beyond the mucosal layer may lead to intestinal wall stiffness, short bowel, colonic dysmotility, and anorectal dysfunction and additionally contribute to increased SF [27]. Our current study included newly diagnosed IBD patients without prior therapy, and PRO symptom subscores showed higher sensitivity, specificity, and positive predictive value with MS in UC patients than in the CD group. Although statistically significant association was found between endoscopic and histopathological activities in UC with patient-reported outcome symptoms, PRO showed inverse correlation with the SES-CD score and histological activity in Crohn's disease patients. Inverse correlation could be explained by the small bowel involvement in the majority of CD patients, with 47 cases of ileocolitis without possible signs of rectal bleeding and normal stool frequency and only 16 patients with CD colitis.

In 2018, a meta-analysis on the diagnostic accuracy of PRO in UC patients with endoscopic remission reported normal values of RB and SF subscores associated with endoscopic remission. Nevertheless, many patients were registered with abnormal stool frequency despite UC endoscopic remission [28]. Patients with persistent symptoms with no, or minimal, bowel inflammation indicate possible high prevalence of irritable bowel syndrome (IBS) in the IBD cohort associated with adverse PRO.

Morris et al. investigated the predictive ability of Crohn's disease activity index (CDAI) for endoscopic disease activity. Although the inclusion of biomarkers, fecal calprotectin, and high sensitivity C-reactive protein improved prediction ability, intraindividual estimation highlighted the PRO model as superior in prediction of CD endoscopic activity [28]. In a large cross-sectional study published in 2016 investigating medication use in IBD patients according to their age and associations with PROs, older CD patients had higher continued steroid use related to worsened patient-reported outcomes [29]. de Jong et al. identified so far more than twenty patient-reported outcome measures (PROMs) used in clinical practice for CD and UC monitoring [30]. The conducted studies emphasized the need of further studies to determine PRO accuracy and utility in the assessment of IBD activity.

## 5. Conclusion

Validated PRO models contribute to a more comprehensive display of IBD burden establishing discrepancies between clinical symptoms and presence of inflammation determined by colonoscopy or histopathology. According to results of our study, PRO-2 correlated with endoscopic and histological activity in UC while inverse correlation was found in CD. Higher sensitivity, specificity, and PPV of PRO-2 were registered in UC. Our results demonstrate that PRO-2 could be used in everyday clinical practice as a simple, noninvasive tool of disease assessment.

## Figures and Tables

**Table 1:** Characteristics of the patients with inflammatory bowel disease.

	UC (*N* = 96)	CD (*N* = 63)	
	*N* (%)	*N* (%)	
Gender (males)	47 (48.9)	40 (63.5)	
Mean age ± SD	42 ± 11	38 ± 11	
Smokers	52 (54.2)	45 (71.4)	
Family history of IBD	1 (1.0)	3 (4.7)	
Appendectomy	2 (2.0)	10 (15.8)	
*Localization of the disease*			
E1 (proctitis)	16 (16.7)	L2 (colonic)	16 (25.4)
E2 (left side)	21 (21.9)	L3 (ileocolonic)	47 (74.6)
E3 (extensive colitis)	59 (61.4)		

Patients with CD without colonic involvement were excluded from the study.

**Table 2:** Impact of UC location on Mayo endoscopic score and Geboes score in study population.

	Location of disease (Montreal)			
UC patients	Proctitis (*n* = 16)	Left side colitis (*n* = 21)	Extensive colitis (*n* = 59)	*p* value
MS (mean ± SD)	1.93 ± 0.85	1.86 ± 0.79	2.05 ± 0.86	*p* = 0.641^a^
Med (range)	2	2	2	
	1-3	1-3	1-3	

Geboes score (mean ± SD)	3.88 ± 1.06	3.64 ± 1.17	3.81 ± 1.32	*p* = 0.595^a^
Med (range)	4.1	4.15	4.20	
	2.1-5.1	2.1-5.1	1.2-5.2	

Statistical significance ^a^Kruskal-Wallis test.

**Table 3:** Correlation of Mayo endoscopic score and histological activity in UC patients.

Mayo score-Geboes score	Spearman's rho (*r*_s_)	*p* value
Overall	0.914	*p* ≤ 0.001^∗^
Proctitis	0.912	*p* ≤ 0.001^∗^
Left-side colitis	0.905	*p* ≤ 0.001^∗^
Extensive colitis	0.910	*p* ≤ 0.001^∗^

^∗^Correlation level was measured using nonparametric Spearman's test and presented with correlation coefficient (r_s_). *p* < 0.05 was considered significant.

**Table 4:** Correlation of SES-CD score and histological activity in CD patients.

CES score-Geboes score	Spearman's rho	*p* value
Overall	0.182	*p* > 0.05
Colitis	0.003	*p* > 0.05
Ileocolitis	0.182	*p* > 0.05

Correlation level was measured using nonparametric Spearman's test and presented with correlation coefficient (*r*_s_). *p* < 0.05 was considered significant.

**Table 5:** Comparison of Mayo endoscopic score to PRO symptom scores.

PRO symptom score	MS (*N* = 96)					
			PPV	NPV	Sensitivity	Specificity
	MS = 1	MS > 1				
RB = 0	28	8	77.8% (64.1-87.3%)	81.6% (72.8-88.1%)	71.8% (55.1-85.0%)	85.9% (74.2-93.7%)
RB > 0	11	49				
SF = 0	24	3	88.9% (71.9-96.1%)	75.6% (68.8-81.3%)	55.8% (39.9-70.9%)	95.2% (86.5-98.9%)
SF > 0	19	50				
RB + SF = 0	25	0	96.0% (77.2-99.4%)	59.1% (53.0-65.1%)	45.3% (31.6-59.5%)	97.7% (87.7-99.4%)
RB + SF > 0	29	42				

PPV: positive predictive value; NPV: negative predictive value. Sensitivity and specificity values are % (95% CI).

**Table 6:** Comparison of SES-CD to PRO symptom subscore.

PRO symptom subscore	SES-CD (*n* = 63)					
			PPV	NPV	Sensitivity	Specificity
	SES ≤ 6	SES > 6				
RB = 0	6	43	12.2% (7.6-19.1%)	71.4% (72.8-88.1%)	60.0% (55.1-85.0%)	78.9% (74.2-93.7%)
RB > 0	4	10				
SF = 0	5	24	28.9% (8.5-31.6%)	75.6% (48.4-68.5%)	26.3% (9.2-51.2%)	45.5% (30.4-61.1%)
SF > 0	14	20				
RB + SF = 0	9	2	81.8% (51.5-95.0%)	40.4% (35.5-45.5%)	22.5% (10.8-38.4%)	97.7% (71.9-98.9%)
RB + SF > 0	31	21				

PPV: positive predictive value; NPV: negative predictive value. Sensitivity and specificity values are % (95% CI).

## Data Availability

The data used to support the findings of this study are available from the corresponding author upon request.
